# Schizophrenia and Suicide Attempts: Findings from a Representative Community-Based Canadian Sample

**DOI:** 10.1155/2016/3165243

**Published:** 2016-02-10

**Authors:** Esme Fuller-Thomson, Bailey Hollister

**Affiliations:** Factor-Inwentash Faculty of Social Work, University of Toronto, Toronto, ON, Canada M5S 1V4

## Abstract

This study examined factors associated with suicide attempts among those with schizophrenia (*n* = 101) versus those without (*n* = 21,643) in a representative sample of noninstitutionalized Canadians. The lifetime prevalence of suicide attempts among persons with schizophrenia was 39.2% versus 2.8% of nonafflicted individuals. After adjusting for sociodemographics, childhood adversities, substance abuse/dependence, depression/anxiety, and chronic pain, those with schizophrenia had 6 times the odds (OR = 6.47) of attempting suicide. Among persons with schizophrenia, suicide attempts were associated with female gender (OR = 4.59), substance abuse/dependence (OR = 6.31), depression (OR = 4.93), and childhood physical abuse (OR = 5.75). Community-dwelling persons with schizophrenia appear to be at high risk for suicide attempts.

## 1. Introduction 

In conjunction with the complexities and difficulties of the symptoms of schizophrenia, individuals with schizophrenia are more likely to attempt suicide than those without schizophrenia [1]. The prevalence of lifetime suicide attempts in those with schizophrenia ranges from 20% to 40% [2–6]. A meta-analysis estimated that one in every 20 individuals with schizophrenia will commit suicide [7]. The average age at suicide is younger among those with schizophrenia than those without the disorder [8]. It is unclear whether men or women with schizophrenia are more likely to attempt suicide with some sources stating that men and women are equally likely [9] while others indicate that women with schizophrenia are more likely to attempt suicide as compared to men with schizophrenia [10]. As will be elaborated below, the direct relationship between schizophrenia and suicide attempts may be attenuated when adjusting for several factors including socioeconomic status, pain, substance use, depression, anxiety, and early adversities.

The evidence concerning the relationship between schizophrenia and socioeconomic status (SES) is mixed [11]. Some studies have demonstrated that growing up in a low income home is associated with increased rates of schizophrenia [12, 13]. In contrast, other studies report an association between growing up in a higher income home and increased rates of schizophrenia [11, 14]. Higher levels of education have been associated with a greater likelihood of suicide completion among those with schizophrenia [15]. The association between social class and suicide completion amongst those with schizophrenia is inconclusive with some studies indicating high social class as a risk factor, while others find homelessness as a risk factor [15].

It is well documented that individuals with schizophrenia experience pain differently compared to the general population [16, 17]. A systematic review found that when there is a clear medical cause for pain, individuals with schizophrenia report a lower prevalence and intensity of pain as compared to the general population [17]. Other studies, both experimental and population-based, support the idea that individuals with schizophrenia experience a decrease in pain sensitivity [16, 18].

A strong association between substance abuse disorders and schizophrenia has been documented [19, 20]. Almost half of Americans with schizophrenia have had some form of substance abuse disorder [21]. A systematic review indicated that for individuals with schizophrenia, alcohol and drug misuse increased the risk of suicide [15].

Symptoms of depression and anxiety are common among individuals with schizophrenia [15, 22, 23]. It is estimated that approximately 50% of individuals with schizophrenia also experience comorbid depression [24]. As in the general population, individuals with schizophrenia who experience depressive symptoms have an increased risk of suicide [5, 15]. A recent systematic review estimated that one in ten (9.8%) of those with schizophrenia had Generalized Anxiety Disorder (GAD) [23]. In the schizophrenia population, individuals with a concurrent social anxiety disorder are more likely to have attempted suicide and to have a greater lethality of suicide attempts than those with schizophrenia without an anxiety disorder [25]. In addition, suicide among those with schizophrenia seems to be less likely a reaction to life events as compared to those without schizophrenia [26].

Early physical, emotional, and sexual abuse, as well as neglect, have been associated with the development of adult onset psychosis and schizophrenia [27–29]. In a systematic review, childhood adversity was indicated as a risk factor for the development of schizophrenia as compared to nonpsychiatric populations [28]. Physical and emotional abuse in childhood are also significantly associated with the development of disassociation symptoms in patients with schizophrenia and psychotic disorders [29]. Patients with schizophrenia who have a history of child maltreatment can experience worse peer relationships, difficulties in school, and more hospital visits at younger ages than those with schizophrenia without a history of child maltreatment [30]. Possibly because of these issues, those with schizophrenia who were maltreated in childhood are more likely than individuals without a history of maltreatment to express intentions of suicide and report depressive symptoms [30]. Childhood trauma in individuals with schizophrenia is associated with an increase in suicide attempts [31, 32], which is similar to findings of studies of the general population [33].

There are several gaps in the current literature. Most studies attempt to understand the relationship between suicidal behaviors and schizophrenia using clinical samples [4, 31, 34, 35]. Studies using representative samples of community dwellers are important too because they can provide information about individuals with schizophrenia who are relatively high functioning, rather than focusing only on those who are institutionalized and tend to be at the more extreme end of the illness continuum. However, the few population-based studies that do exist often use data from outside of North America [36, 37]. One Canadian population-based study found that schizophrenia is associated with an increased risk of suicide, particularly within the first 90 days of diagnosis [38]. An increased risk of suicide in those with schizophrenia has also been demonstrated immediately after discharge from hospitals [39].

A few population-based studies have been conducted with those diagnosed with schizophrenia outside of North America. For example, a Danish cohort study found that low birth weight, low IQ, and low socioeconomic status are associated with the development of psychiatric illnesses as well as drug and alcohol abuse in men later in life [40]. This drug and alcohol abuse was related to poorer survival rates [40]. A population-based study in Denmark found that younger people (<35) were most at risk for suicide as compared to other age categories [41]. This was true for both genders [41]. A Swedish population-based study concerning violent behavior and suicide indicated that, for men, alcohol use, drug use, previous self-harm, and previous violent offenses all increased suicide risk [42]. For women, self-harm was found to be associated with an increased suicide risk [42].

Another gap in the literature is that most studies analyze suicide risk, suicidal ideation, or suicide in general [1, 15, 43] and, thus, there is less known about suicide attempts, which are strongly associated with both future attempts and completed suicides [1, 3, 34]. To our knowledge, no studies have examined the role of witnessing parental domestic violence and its relationship to schizophrenia and suicide attempts. As witnessing domestic violence has been associated with several negative outcomes in adults, research to better understand its role and relationship to schizophrenia and suicide attempts is needed [44]. Our hypotheses are as follows: (1) noninstitutionalized people with schizophrenia will have higher odds of suicide attempts than those without the disorder, and this association will be partially attenuated when adverse childhood experiences, sociodemographics, and mental health and substance abuse are accounted for in the analysis and (2) among community-dwellers with schizophrenia the odds of suicide attempts will be associated with each of the three types of childhood adversity (childhood physical abuse, childhood sexual abuse, and witnessing parental domestic violence), substance abuse, depression, and anxiety disorders.

## 2. Methods

Data from the 2012 Canadian Community Health Survey-Mental Health (CCHS-MH) were analyzed in the current study. The CCHS-MH is a cross-sectional survey that collects information about the factors, influences, and processes that contribute to mental health through a multidisciplinary approach focusing on health, social, and economic determinants [45]. While the survey covers the population of individuals at age of 15 and older living in the 10 provinces, residents of the three territories, those living on reserves or other aboriginal settlements, full-time members of the armed forces, and the institutionalized population were excluded. The latter groups represent less than 3% of the target population [45].

### 2.1. Sample

Respondents for the CCHS-MH were sampled using a three-stage design, which began with selecting individuals from geographical areas and then households within each geographical area. Out of all eligible respondents, one individual within each household was randomly selected according to varying selection probabilities [45]. Based on the selected units, 36,443 were in-scope for the survey and, of these, 29,088 households agreed to participate in the survey, resulting in an overall household-level response rate of 79.8%. From responding households, 29,088 persons were selected to participate and 25,113 provided a valid questionnaire, which yielded an overall person-level response rate of 86.3%. At the national level, these figures result in a combined response rate of 68.9%.

The analysis for the current study is based on two subsamples of the full CCHS-MH. More specifically, the first analysis was restricted to those with complete data on each of the measures in the analyses (*n* = 21,744). It only contains respondents at age of 20 and older because younger respondents were not asked questions about adverse childhood experiences. The second analysis was a subsample of the first analysis restricted to respondents who reported that they had been diagnosed with schizophrenia (*n* = 101).

### 2.2. Measures

*Schizophrenia* was assessed based upon a list of “conditions diagnosed by a health professional that are expected to last or have already lasted 6 months or more.” Those with a positive response to the question “do you have schizophrenia?” were coded as such.

*Suicide attempt* was ascertained by a positive response to the question: Have you ever “attempted suicide or tried to take your own life?” The questions were found in a section of the CCHS-MH that focuses specifically on suicide and beginning with a statement that “the questions may be sensitive to some people, but we have to ask the same questions of everyone.”

*Sociodemographic variables* include gender, self-identified race (nonaboriginal White versus visible minority and/or aboriginal), age (in decades), and* Socioeconomic Position* (SEP) measured by the highest level of achieved education (less than high school, high school graduate, some postsecondary school, postsecondary diploma, or university degree). Statistics Canada's measure of household income as a ratio related to the national low-income cut-off, divided into deciles, was also used to assess SEP. This variable takes into account the number of people in the household and the size of the community.

*Adverse Childhood Experiences (ACEs)* included the following. 

*(i) Witnessing Parental Domestic Violence (PDV)*. Individuals were determined as having been exposed to intimate partner violence if they reported they had seen or heard 11 or more times one of their “parents, step-parents, or guardians hit each other or another adult” aged 18 or over in their home.

*(ii) Childhood Sexual Abuse (CSA)*. Childhood sexual abuse was measured by the question “How many times did an adult force you or attempt to force you into any unwanted sexual activity, by threatening you, holding you down, or hurting you in some way?” This variable was coded never versus ever.

*(iii) Childhood Physical Abuse (CPA)*. Individuals were ascertained as having been physically abused if they reported that, before they reached the age of 16, an adult had at least once kicked, bit, punch, choked, burned, or physically attacked them.

*Mental health factors* include lifetime anxiety and depression. Individuals were coded as having an anxiety disorder if they met the WHO-CIDI lifetime criteria for Generalized Anxiety Disorder. Similarly, respondents were ascertained as a depressive disorder if they met the WHO-CIDI lifetime criteria for Major Depressive Episode. For more details please see [45].

*Chronic pain* was assessed in a section of the survey focusing on pain and discomfort that began with the following statement: the next set of questions asks about the level of pain or discomfort you usually experience. They are not about illnesses like colds that affect people for short periods of time. Respondents were determined to be regularly in pain if they responded no to the question “Are you usually free of pain or discomfort?”

*Substance abuse* was measured based on the WHO-CIDI using a combination of the derived variables created by Statistics Canada “Drug Abuse or Dependence (including Cannabis)” and “Alcohol Abuse or Dependence.” Both were derived using the lifetime algorithm. For more information, please see the Canadian Community Health Survey (CCHS) mental health derived variable specifications [45].

### 2.3. Statistical Analyses

The lifetime prevalence of suicide attempts among those with schizophrenia was calculated in SPSS 22. Chi-square analysis was conducted to examine gender difference in the lifetime prevalence of suicide attempts among those with and without schizophrenia. Two series of logistic regression analyses were conducted. In the first series of analyses, the outcome was lifetime suicide attempt and the key exposure variable was schizophrenia status. The goals of the analyses were to document the odds of suicide attempts among those with schizophrenia in comparison to those without and to calculate the degree to which inclusion of the potentially confounding factors attenuated the relationship. Age, race, income, and education were controlled for in the first model and each subsequent model. The second and third model adjusted for three early adversities (witnessing parental domestic violence, being physically abused, and being sexually abused as a child) and chronic pain, respectively. The fourth model controlled for drug and alcohol abuse. The fifth model accounted for lifetime depression and anxiety disorders. The sixth and final model controlled for all the above-named factors simultaneously.

A second series of logistic regression analyses was completed using the subsample of those diagnosed with schizophrenia (*n* = 101). The first model controlled for childhood adversities only, the next adjusted for gender and substance abuse/dependence, and a third accounted for mental health factors. A final model controlled for all the above-named variables simultaneously. Data from all the analyses were weighted to adjust for the probability of selection and nonresponse. Sample sizes are reported in their unweighted form. Odds ratios and 95% confidence intervals are reported.

## 3. Results

In our population-based sample of noninstitutionalized Canadians, the lifetime prevalence of suicide attempts among those with schizophrenia was 39.2% compared to 2.8% of individuals without schizophrenia. One-third of males (33%) with schizophrenia had attempted suicide in contrast to 2.2% of males without schizophrenia (*p* < 0.001). Almost half of women (47.3%) with schizophrenia had attempted suicide in comparison to 3.5% of women without schizophrenia (*p* < 0.001).

When the data were adjusted for age, race, income, and education, noninstitutionalized individuals with schizophrenia had 15 times the odds of attempting suicide (OR: 15.74, 95% CI = 9.84, 25.2) in comparison to those without schizophrenia (please see Table 1). We conducted a series of logistic regression analyses all of which included the above variables in addition to several different clusters of risk factors that we anticipated would attenuate the association between schizophrenia and suicide attempts. When three early adversities (witnessing parental domestic violence, being physically abused, and being sexually abused as a child) were added to the equation, the odds of suicide attempts among those with schizophrenia declined to 12.92 (OR: 12.92, 95% CI = 7.96, 21.753). When drug and alcohol abuse were included, people with schizophrenia still had 13 times the odds (OR: 13.06) of attempting suicide as compared to individuals without schizophrenia. When pain was added to the sociodemographic variables, people with schizophrenia had 14.5 times the odds of attempting suicide (OR: 14.5, CI = 8.91, 23.74). The greatest attenuation in the schizophrenia-suicide attempt association was due to the inclusion of lifetime depression and anxiety disorders. Inclusion of these mental health characteristics reduced the odds of suicide attempts among those with schizophrenia to 7.22 (95% CI = 4.22–12.36). After adjusting for all of the factors discussed above, individuals with schizophrenia had 6 times the odds (OR: 6.47) of attempting to suicide as compared to individuals who did not have schizophrenia. Among the variables controlled for, mental health factors explained the largest share of the relationship between schizophrenia and suicide attempts at 58%, followed by childhood adversities at 20% and substance use at 18%. All the factors together accounted for 63% of the association.

In order to identify the key factors associated with suicide attempts among those with schizophrenia, we conducted additional analyses restricted to the 101 respondents in the CCHS-MH data set who reported that they had been diagnosed with schizophrenia by a medical professional (please see Table 2). In a model adjusting for all variables simultaneously, we found that suicide attempts were associated with female gender (OR = 4.59), substance dependence (OR = 6.31), depression (OR = 4.93), and a history of physical abuse in childhood (OR = 5.75). When only early adversities were included, exposure to chronic parental domestic violence was associated with almost 7 times the odds of suicide attempts (OR = 6.95). After adjustments for gender, substance abuse, and depression and anxiety, this number decreased to nonsignificance (OR: 2.58, 95% CI = 0.65, 10.30). Among those with schizophrenia, early adversity accounted for 24% of the variability in suicide attempts (Nagelkerke *R* ^2^ = 0.24). When gender, drug and alcohol abuse, and lifetime anxiety and depressive disorders were added to the analysis, 38% of the variability in suicide attempts was accounted for (Nagelkerke *R* ^2^ = 0.38).

## 4. Discussion 

The purpose of this study was to examine the relationship between suicide attempts and schizophrenia using representative community-based data. In individuals with schizophrenia, 39.2% had attempted suicide as compared to 2.8% of individuals without schizophrenia. Previous studies have estimated the prevalence of suicide attempts in individuals with schizophrenia to range from 20 to 40% [2–6]. Although the percentage of individuals with schizophrenia who attempted suicide fell between previously estimated rates, it was theorized that, with population-based data of noninstitutionalized individuals, these numbers would be lower than in studies using clinical samples. The data suggest, however, that rates of suicide attempts in those with schizophrenia living within the community may be similar to those found in clinical settings.

We found that the odds of suicide attempt were over 6 times higher among those with schizophrenia compared to those without, even when accounting for lifetime depression and anxiety disorders, substance use, childhood adversities, pain, and sociodemographics. Among the latter factors, depression and anxiety explained the largest share of the relationship between schizophrenia and suicide attempts, which is consistent with research showing that as much as half of individuals with schizophrenia suffer from depressive symptoms and that these symptoms are associated with an increase in suicide attempts [5, 15, 24]. Among those with schizophrenia, we found that depression was associated with 7 times the odds of a suicide attempt and that depression and anxiety together explained 17.5% of the variability in suicide attempts. These findings suggest that it is important for health care professionals working in the community to be aware of the need to assess for the presence of other mental health problems alongside the symptoms of schizophrenia. Developing best practices to support individuals who are experiencing schizophrenia comorbid with other mental health conditions could help decrease the prevalence of suicide attempts among those living in the community.

Interestingly, in the fully adjusted model, those with an anxiety disorder were 69% less likely to attempt suicide. Rapp et al. [46] found that individuals with schizophrenia who also had anxiety demonstrated higher verbal IQ, better problem solving, attention, and verbal fluency. Possessing these qualities might indicate that individuals with both schizophrenia and anxiety may be more likely to have the necessary skills to express themselves verbally and communicate with those around them should they be experiencing suicidal ideation, increasing the chances of a successful intervention. More research is required to understand the differences in characteristics between those with schizophrenia with an anxiety disorder and those with schizophrenia without an anxiety disorder.

In the general population, women are more likely to attempt suicide than men [47]. Some research has also identified this trend for women with schizophrenia [10], which is in keeping with what we found in our data. When only sociodemographics and substance abuse history were included in the analysis, women's odds of suicide attempts were elevated but failed to reach statistical significance (OR = 2.13; 95% CI = 0.91, 4.98). However, these odds increased and became statistically significant when histories of depressive disorders and anxiety disorders were included (OR = 3.10; 95% CI = 1.19, 8.04). In the final model we were surprised by the magnitude of the association in our sample: women with schizophrenia had four times the odds of suicide attempts compared to men with schizophrenia when adjustments were made for adverse childhood experiences, sociodemographics, substance abuse, depression, and anxiety disorder (OR = 4.59; 95% CI = 1.21, 17.35). It appears that women with schizophrenia are particularly at risk, even independent of these comorbid conditions. One potential factor to explain this is that females with schizophrenia tend to present with symptoms later than males [10, 47], and individuals with later onset schizophrenia have been shown to have an increased risk of suicide [48, 49].

Among those with schizophrenia, 24% of the variability in suicide attempts was explained by a history of early adversity. When only early adversities were used to predict suicide attempts among those with schizophrenia, those who had been exposed to chronic parental domestic violence had approximately seven times the odds of attempting suicide. To our knowledge, this is the first study to examine the role of exposure to parental domestic violence in those with schizophrenia and its relationship to suicide attempts. Our findings are in keeping with data from the general population which has shown that individuals who are exposed to parental domestic violence have an increased risk of suicide attempts [50]. Childhood physical abuse was associated with 2.66 higher odds of suicide in those with schizophrenia; however, it only reached borderline significance (model 1: *p* < 0.065). After adjustments for demographics, substance abuse, and history of depression and anxiety disorders, the relationship between parental domestic violence and suicide attempts decreased to nonsignificance but childhood physical abuse reached statistical signficance. Our findings are in contrast to a recent clinical study of suicide attempts before and after first episode of schizophrenia which found no association between childhood traumas and suicide attempts [51]. There were numerous design differences between Togay et al.'s study [51] and this study which may have led to the different findings including source of sample (clinical sample of first episode clients versus representative population-based study), study location (Turkey versus Canada), period of suicide attempts (average of 5 years after first episode versus lifetime), and different measures of early adversities (combined measure of 5 types of abuse using a standardized scale versus separate inclusion of 3 specific types of childhood adversities). Physical, emotional, and sexual abuse, neglect, and witnessing parental domestic violence have been associated with an increased risk in suicide attempts in the general population [31, 32]. In our Canadian study, early childhood adversities explained a large portion of the variability in suicide attempts among those with schizophrenia. These findings reinforce the need to develop targeted programs for individuals with schizophrenia who have experienced early childhood abuse [31, 32].

In the fully adjusted model, substance abuse/dependence was associated with 6 times the odds of suicide attempts among those with schizophrenia. Both in the general population and in the schizophrenia literature drug abuse and addictions have been associated with increased risk of suicide attempts [19, 20, 52, 53]. Interestingly, substance abuse/dependence explained just 7% of the variation in suicide attempts. A potential explanation for the finding rests in exploring the self-medication hypothesis [54]. It is theorized that part of the reason individuals with schizophrenia have such high rates of substance abuse is that they try to alleviate the pain and suffering they feel as a result of the symptoms of schizophrenia by using substances [54, 55]. It is possible that, due to our use of population-based data of noninstitutionalized individuals, our sample may not be experiencing the same level of intensity of symptoms as one would see in a clinical sample and, thus, is less likely to be using substances to cope and, subsequently, turn to attempting suicide.

There are a several limitations present in this study. It is possible that recall bias may have occurred considering the retrospective nature of reporting suicide attempts as well as early adversities. There has been a lack of research aimed at establishing the validity and reliability of self-report measures related to suicide attempts [56]. In the general population, the evidence concerning the validity and reliability of retrospective reporting of child maltreatment is mixed, with some studies finding the self-reports an accurate way of assessing child maltreatment [57], while others are finding significant instability in retrospective reports of child maltreatment [58]. In individuals with schizophrenia, concerns about memory and cognition could be cited as a potential challenge to relying on self-report data [59]. Although slower recall and deficits in recalling emotion laden memories have been reported in individuals with schizophrenia, most of these studies have relied on clinical samples, thus potentially skewing the understanding of memory deficits in those with schizophrenia [60–62]. Other research has found good validity in self-report measures of childhood abuse among patients with mental disorders [63]. There are some potential risk factors for suicide attempts in individuals with schizophrenia that were not included in our analysis. These include insight into disease, poor adherence to treatment, schizophrenia symptom profile, housing conditions, marital status, and living alone. The absence of these important potential confounds could have biased our findings.

Questions have also been raised as to whether self-report of a medical diagnosis of schizophrenia is a reasonable method to assess the presence or absence of this condition. Self-reports of a medical diagnosis of schizophrenia are a prominent technique used in large, population-based data sets [64]. The prevalence of schizophrenia based on self-report of a medical diagnosis in the current sample was in keeping with the systematic review by Saha and colleagues [65] which estimated that the lifetime prevalence of schizophrenia was approximately 0.44%. However, alternative techniques for assessing schizophrenia status, such as screening tools and self-report measures, also carry the risk of missing cases of psychiatric illnesses [66]. There is particularly the potential for missing those with negative symptoms [66, 67]. In a previous study using an earlier wave of the CCHS, self-report of a clinical diagnosis of schizophrenia was found to be a reasonable way to assess the existence of schizophrenia [64]. There have been few studies examining this phenomenon, and it has been suggested that more research is to be conducted using diagnostic interviewing as a comparison to produce better, more reliable data [64]. Although the overall response rate of 68.9% was quite good for a large population-based survey, it is possible that those with schizophrenia, particularly those with more severe symptoms, may be less likely to participate in a survey such as this. If the most severe cases are most likely to have had suicide attempts, it is likely that such an underrepresentation of these individuals would result in our analyses underestimating the magnitude of the relationship between schizophrenia and suicide attempts. Additionally, it is possible that the question* “How many times did an adult force you or attempt to force you into any unwanted sexual activity, by threatening you, holding you down, or hurting you in some way?" *does not adequately capture all types of sexual abuse, specifically forms that would be subtler in nature. Consequently, our study might underestimate the history of sexual abuse.

Our nationally representative sample suggests noninstitutionalized individuals with schizophrenia are 6 times more likely than individuals without schizophrenia to attempt suicide. The presence of depressive symptoms, as well as early childhood adversities, played significant roles in explaining the high rates of suicide within the schizophrenia population. With this information, it is important to recognize that individuals who are experiencing psychosis are also vulnerable to other serious mental health issues, which are related to suicide attempts. This, alongside targeted programs for those with schizophrenia who have experienced early adversity, could help reduce the suicide attempts by individuals with schizophrenia living in the community.

## Figures and Tables

**Table 1:** Odds ratios and 95% confidence interval of lifetime suicide attempts among those with schizophrenia versus those without in a representative community-based sample (*n* = 21744).

	Model 1	Model 2	Model 3	Model 4	Model 5	Model 6
	Demographics + SES	Demographics + SES + ACEs	Demographics + SES + substance abuse	Demographics + SES + mental health	Demographics + SES + pain	Fully adjusted
Has schizophrenia						
Yes	15.74 (9.84, 25.18)	12.92 (7.68, 21.75)	13.06 (7.95, 21.47)	7.23 (4.22, 12.36)	14.54 (8.91, 23.74)	6.47 (3.73, 11.20)
No (Ref)	1.00	1.00	1.00	1.00	1.00	1.00
Adverse childhood experiences						
Witnessed parental domestic violence						
>10 times	—	2.40 (1.88, 3.06)	—	—	—	2.21 (1.71, 2.86)
≤10 times (Ref)	—	1.00	—	—	—	1.00
Physical abuse						
Abused	—	3.35 (2.72, 4.13)	—	—	—	2.28 (1.84, 2.83)
None (Ref)	—	1.00	—	—	—	1.00
Sexual abuse						
Abused	—	4.21 (3.41, 5.21)	—	—	—	2.85 (2.28, 3.56)
None (Ref)	—	1.00	—	—	—	1.00
Demographics						
Sex						
Female	1.57 (1.33, 1.85)	1.44 (1.21, 1.72)	2.33 (1.95, 2.77)	1.25 (1.05, 1.48)	1.50 (1.27, 1.77)	1.49 (1.23, 1.80)
Male (Ref)	1.00	1.00	1.00	1.00	1.00	1.00
Race						
White only	1.33 (1.09, 1.61)	1.31 (1.07, 1.61)	0.97 (0.79, 1.19)	0.99 (0.81, 1.22)	1.22 (1.01, 1.49)	0.94 (0.76, 1.17)
Visible minority or aboriginal (Ref)	1.00	1.00	1.00	1.00	1.00	1.00
Age						
Age by decade	0.82 (0.78, 0.86)	0.80 (0.76, 0.84)	0.88 (0.84, 0.93)	0.84 (0.79, 0.88)	0.77 (0.73, 0.81)	0.82 (0.77, 0.87)
Socioeconomic status						
Education						
No high school graduation (Ref)	1.00	1.00	1.00	1.00	1.00	1.00
High school graduate	0.64 (0.50, 0.83)	0.73 (0.56, 0.96)	0.64 (0.50, 0.83)	0.58 (0.45, 0.76)	0.69 (0.54, 0.90)	0.64 (0.48, 0.85)
Postsecondary graduate	0.79 (0.64, 0.99)	0.93 (0.74, 1.18)	0.87 (0.70, 1.09)	0.73 (0.58, 0.91)	0.87 (0.70, 1.09)	0.93 (0.73, 1.19)
Household income (decile)	0.84 (0.81, 0.87)	0.87 (0.85, 0.90)	0.83 (0.80, 0.86)	0.86 (0.84, 0.89)	0.86 (0.83, 0.89)	0.89 (0.86, 0.92)
Drug or alcohol abuse						
Yes	—	—	5.57 (4.68, 6.64)	—	—	2.77 (2.29, 3.34)
No (Ref)	—	—	1.00	—	—	1.00
Mental health						
Lifetime generalized anxiety disorder						
Yes	—	—	—	3.23 (2.66, 3.93)	—	2.13 (1.73, 2.63)
No (Ref)	—	—	—	1.00	—	1.00
Lifetime depression						
Yes	—	—	—	5.43 (4.51, 6.55)	—	3.85 (3.17, 4.68)
No (Ref)	—	—	—	1.00	—	1.00
Chronic pain						
Pain						
Moderate or severe	—	—	—	—	4.13 (3.46, 4.93)	1.82 (1.49, 2.22)
Less than moderate (Ref)	—	—	—	—	1.00	1.00

Nagelkerke *R* square value	0.070	0.193	0.140	0.201	0.112	0.303
−2 Log likelihood	5431.6	4787.3	5068.4	4747.6	5213.9	4194.1

SES = socioeconomic status, ACEs = adverse childhood experiences.

**Table 2:** Odds ratios and 95% confidence interval of lifetime suicide attempt among those with schizophrenia from a representative community-based sample (*n* = 101).

	Model 1	Model 2	Model 3	Model 4
	ACEs only	Demographics and substance abuse	Demographics, substance abuse, and mental health	Fully adjusted
Adverse childhood experiences				
Witnessed parental domestic violence				
>10 times	6.95 (2.30, 20.99)	—	—	2.58 (0.64, 10.30)
≤10 times (Ref)	1.00	—	—	1.00
Physical abuse				
Abused	2.66 (0.94, 7.51)	—	—	5.75 (1.53, 21.58)
None (Ref)	1.00	—	—	1.00
Sexual abuse				
Abused	1.00 (0.33, 3.04)	—	—	0.45 (0.13, 1.61)
None (Ref)	1.00	—	—	1.00
Demographics				
Sex				
Female	—	2.13 (0.91, 4.98)	3.10 (1.19, 8.04)	4.59 (1.21, 17.35)
Male (Ref)	—	1.00	1.00	1.00
Drug or alcohol abuse				
Yes	—	2.24 (0.96, 5.22)	5.02 (1.77, 14.26)	6.31 (1.83, 21.72)
No (Ref)	—	1.00	1.00	1.00
Mental health				
Anxiety				
Lifetime anxiety disorder	—	—	0.37 (0.13, 1.02)	0.31 (0.10, 0.94)
No anxiety disorder (Ref)	—	—	1.00	1.00
Depression				
Lifetime depressive disorder	—	—	6.97 (2.37, 20.52)	4.93 (1.26, 19.28)
No depression (Ref)	—	—	1.00	1.00

Nagelkerke *R* square value	0.241	0.074	0.250	0.381
−2 Log likelihood	115.5	129.629	114.7	101.9

ACE: adverse childhood experience.
